# Plasma Biomarkers Associated with Incident Mobility, Cognitive, and Dual Impairment in Older Adults: the ARIC Study

**DOI:** 10.1093/geroni/igaf122.3364

**Published:** 2025-12-31

**Authors:** Jayanti Shukla, Chad Blackshear, David Knopman, Priya Palta, Michael Griswold, B Gwen Windham, Thomas Mosley, Kevin Sullivan

**Affiliations:** University of Mississippi School of Medicine, Jackson, Mississippi, United States; University of Mississippi Medical Center - The MIND Center, Jackson, Mississippi, United States; Mayo Clinic, Rochester, Minnesota, United States; UNC School of Medicine, Chapel Hill, North Carolina, United States; University of Mississippi Medical Center - The MIND Center, Jackson, Mississippi, United States; University of Mississippi Medical Center - The MIND Center, Jackson, Mississippi, United States; University of Mississippi Medical Center - The MIND Center, Jackson, Mississippi, United States; University of Mississippi Medical Center - The MIND Center, Jackson, Mississippi, United States

## Abstract

Mobility and cognition impairments are common in older adults, but neurodegenerative pathology contributions are unclear. We examined cross-temporal associations of Alzheimer’s Disease (AD)-specific and nonspecific neurodegenerative plasma biomarkers neurofilament light (NfL) with incident mobility and cognitive impairment in the Atherosclerosis Risk in Communities (ARIC) study. We hypothesized NfL would show stronger associations with mobility impairment than p-tau181 in a subset of 1,227 cognitively normal participants with biomarker data and no mobility impairment (mean age=75.5±5.0, 56% female, 23% Black) at visit 5 (2011-13). Participants were classified into five functional groups using data from up to four exams: no impairment (n = 587; referent), mobility impairment only (n = 266; gait speed< 0.8m/s), cognitive impairment only (n = 69; adjudicated dementia), dual impairment (n = 243; both) or decline-free death (n = 262) over a mean 6.3 years (0-11 years) of follow-up. A multinomial regression model estimated association of functional groups with p-tau-181 and NfL jointly, adjusted for cardiovascular and demographic factors, BMI, and kidney function. Relative prevalence ratios (RPR; 95% CI) are presented per one-unit higher log-base-2 transformed biomarker (doubling). For mobility and cognitive functional outcomes, p-tau-181 was more robustly supported [Mobility: RPR=1.36, (1.04,1.77); Cognitive: RPR=1.73, (1.10,2.70); Dual: RPR=1.78, (1.15,2.77)] than NfL [Mobility: RPR=1.14, (0.85,1.53); Cognitive: RPR=1.20, (0.70,2.05); Dual: RPR=1.65, (0.86,3.16)]; higher pTau-181 [RPR=1.60, (1.23,2.09)] and higher NfL [RPR=1.94, (1.39,2.71)] were associated with higher risk of decline-free death. A p-tau-181-x-NfL interaction term was not supported. Findings did not support NfL associations with functional outcomes with consideration of p-tau-181. AD blood biomarkers may help identify individuals at greater risk for multifaceted disability.

